# Unveiling the Impact of Drug-Sensitive Mutations on HIV-1 Protease Dynamics: A Molecular Dynamics Simulation Study of the T12A, L63Q, and H69N Variants

**DOI:** 10.3390/ijms27093832

**Published:** 2026-04-25

**Authors:** Haythem Srihi, Nabil Abid, Lavinia Fabeni, Caterina Precone, Hélène Déméné, Giovanni Chillemi

**Affiliations:** 1 Research Unit UR17ES30 “Genomics, Biotechnology and Antiviral Strategies”, High Institute of Biotechnology of Monastir, University of Monastir, Tahar Hadded Avenue, PB 74, Monastir 5000, Tunisia; srihi.haythem@gmail.com; 2 High Institute of Biotechnology of Monastir, Department of Molecular and Cellular Biology, University of Monastir, Tahar Hadded Avenue, BP 74, Monastir 5000, Tunisia; nabilabidbensalem.2014@yahoo.fr; 3 Laboratory of Transmissible Diseases and Biological Active Substances LR99ES27, Faculty of Pharmacy, University of Monastir, Rue Ibn Sina, Monastir 5000, Tunisia; 4 Laboratory of Virology, National Institute for Infectious Diseases Lazzaro Spallanzani—IRCCS, Via Portuense, 292, 00149 Rome, Italy; lavinia.fabeni@inmi.it; 5 Department for Innovation in Biological, Agro-Food and Forest Systems—DIBAF, University of Tuscia, 01100 Viterbo, Italy; caterina.precone@unitus.it; 6 Centre de Biochimie Structurale (CBS), INSERM, CNRS, Université de Montpellier, 29 Rue de Navacelles, 34090 Montpellier, France; 7 Bioinformatics Research Unit in Infectious Diseases, National Institute for Infectious Diseases Lazzaro Spallanzani—IRCCS, Via Portuense, 292, 00149 Rome, Italy; 8 Department of Experimental Medicine, University of Rome “Tor Vergata”, Via Montpellier 1, 00133 Rome, Italy

**Keywords:** HIV-1 protease, protease inhibitors, long range protein conformation, molecular dynamics simulations

## Abstract

HIV-1 protease (PR) is an essential enzyme in the viral life cycle and a primary target of antiretroviral therapies, particularly protease inhibitors (PIs). Understanding the dynamics of viral evolution and the factors governing the emergence or loss of resistance-associated mutations is critical for improving PI efficacy and managing drug resistance in HIV/AIDS treatment. In this study, we investigated the impact of three natural HIV-1 polymorphisms (T12A, L63Q, and H69N), whose prevalence varies depending on treatment status and viral subtype, on the structural stability and conformational dynamics of PR using molecular dynamics (MD) simulations. Three independent 500 ns MD simulations were performed for the native protease and each mutant system. Although none of the mutations disrupts the overall structural integrity of HIV-1 PR, they induce mutation-specific alterations in flexibility and residue interactions. In particular, T12A and H69N exhibit increased structural deviations, especially in the flap regions, along with enhanced conformational fluctuations. In contrast, the L63Q mutation shows a slight reduction in flap flexibility compared to both the native protease and the other mutants. Consistently, the fraction of time spent in open-flap conformations is higher for T12A and H69N and lower for L63Q relative to the native system. Moreover, mutations in the Fulcrum (T12A) and Cantilever (L63Q and H69N) regions do not disrupt the long-range network of correlated motions observed in the native protease, both inter- and intra-monomer, but instead increase the extent of correlated and anti-correlated motions in other regions of PR.

## 1. Introduction

The evolution of the HIV-1 genome and the emergence of drug-resistant viral variants remain major challenges for the scientific community. HIV resistance is defined as the virus’s ability to replicate in the presence of one or more inhibitory molecules. This resistance can be attributed to the error-prone nature of HIV-1 reverse transcriptase (RT), which induces several mutations during virus replication, leading to drug-resistant properties [1].

To date, several classes of antiretroviral drugs are used in the treatment of HIV infection. Each class targets different stages of the HIV life cycle, including virus entry and fusion with the host cell, as well as viral enzymes like protease, reverse transcriptase, and integrase. Currently combination antiretroviral therapy, which includes nucleoside and non-nucleoside RT inhibitors, as well as entry, integrase, in addition to protease inhibitors (PIs), has significantly reduced viral load and improved the life expectancy of people living with HIV (PLH) [2,3]. Indeed, HIV protease (PR) is a crucial drug target for HIV/Acquired Immuno Deficiency Syndrome (AIDS) therapy, and PIs have significantly improved patient outcomes since their introduction in 1995 [4]. HIV-1 PR plays an essential role in viral maturation by processing specific cleavage sites in the Gag and Gag-Pol precursor polyproteins to release mature proteins. This protease-catalyzed processing is essential for forming infectious virus particles [5,6,7]. The structures and activities of PR, along with its drug-resistant variants and their interactions with inhibitors, have been studied extensively to address the challenges of AIDS antiviral therapy and the evolution of HIV drug resistance [8,9,10,11,12,13].

Despite the success of combination antiretroviral therapy, the persistence of viral reservoirs and the continuous emergence of resistant variants remain major obstacles to long-term viral suppression [8,10]. In this context, structural and dynamical investigations of HIV-1 protease are crucial not only for understanding resistance mechanisms but also for guiding the design of next-generation inhibitors with improved robustness against mutational escape.

**Figure 1:**
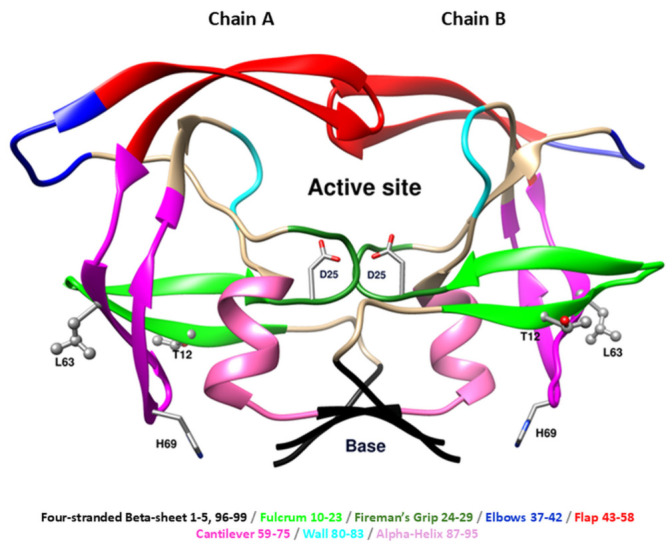
Topology of the HIV-1 protease (PDB ID: 1ODW) in dimeric form. Topology of HIV-1 protease colored according to the convention proposed previously (Sadiq and De Fabritiis, 2010) [14]. The three mutated residues T12, L63, and H69 are shown in ball and stick representation. The catalytic residue D25 is shown in stick representation.

Several terminologies were used to describe the topology of HIV protease according to some mechanistic hypothesis. In this article we adopted the one introduced by De Fabritiis’ group [14]. The dimer interface is formed principally from an interdigitated four-stranded beta-sheet (residues 1–5, 96–99) and the so-called fireman’s grip (residues 24–29) that provide the base for the active site and entails the catalytic Asp dyad (D25). The active site is enclosed on top by flexible β-hairpin structure called the ‘‘flap’’ (residues 43–58) and laterally by the ‘‘wall’’ (residues 80–83). Additional features of each monomer are the flap elbows (residues 37–42), the cantilever (residues 59–75), the fulcrum (residues 10–23), and the α-helix (residues 87–95) (Figure 1). It was shown that the PR dimer adopts a closed conformation when bound to a substrate or inhibitor, whereas flap opening modulates the entry or release of the ligands [15].

PI-resistance mutations are associated with the highest levels of phenotypic resistance and/or the strongest clinical evidence for interfering with successful PI therapy (https://hivdb.stanford.edu/dr-summary/resistance-notes/PI/, access date on 23 January 2026). It has been reported that three PR mutations located in the fulcrum (T12) and cantilever domains (L63 and H69) [16,17,18] are categorized as polymorphisms—mutations usually observed more frequently in drug-naïve individuals and typically reduced in prevalence among patients treated with protease inhibitors (PIs) [18,19]. These mutations, although naturally occurring, tend to be negatively selected in PI-treated patients, suggesting a potential compensatory or modulating role. Epidemiological data from the Stanford HIV Drug Resistance Database indicates that T12A, L63Q, and H69N are not primary resistance mutations but may influence protease dynamics and drug susceptibility indirectly. Their prevalence varies depending on treatment status and viral subtype, and they may transiently emerge or disappear under drug pressure, even if the exact role of these mutations in protease structure has not yet been precisely clarified. To this end, molecular dynamics (MD) simulations were used to simulate protein movements at atomic resolution for the three discussed mutants as well as the native form of HIV protease protein, in three replicas. The resulting structural data were analyzed to investigate the effects of these mutations on protein structure, dynamics, and its ability to function properly.

## 2. Results

### 2.1. Stability of the HIV-1 PR Systems

Our study first tested the equilibrium state and overall stability of the different protein structures in their apo state along the generated trajectories, using the root-mean-square deviation (RMSD) analysis. All simulations were initiated from energy-minimized closed conformations and simulated for 500 ns.

**Figure 2:**
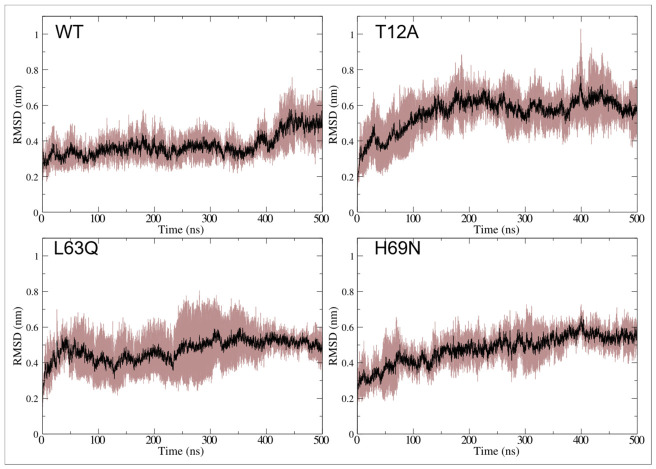
Plot of RMSD of c-alpha atoms, as a function of simulation time for the four simulated PR systems. Average and shaded error range, calculated from the three replicas, are colored in black and brown, respectively.

The average RMSD values and corresponding shaded error ranges, calculated from the three replicas, are shown in Figure 2 for the four systems. The three replicas of the wild-type (WT) exhibit good reproducibility, whereas greater variability is observed among the mutant replicas. Individual RMSD profiles for each replica are reported in Supplementary Figure S1. All systems sample conformations with RMSD values greater than 0.7 nm relative to the initial MD structure. Average, standard deviation and maximum RMSD values for R1, R2 and R3 across all systems are reported in Table 1.

The T12A mutant exhibits higher RMSD values than all other PR systems. However, all mutations lead to increased RMSD values across replicas, indicating an intrinsic effect on the conformational space sampled by PR.

**Figure 3:**
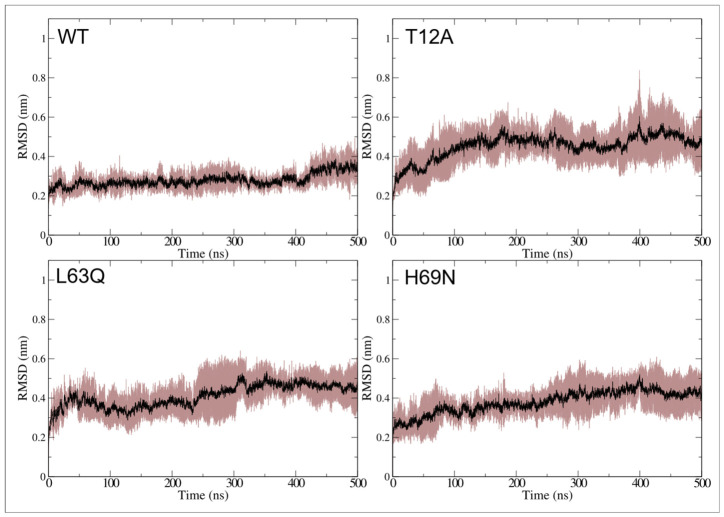
Plot of RMSD of c-alpha atoms with the exclusion of the flap domains residues (43–58), as a function of simulation time for the four simulated PR systems. Average and shaded error range, calculated from the three replicas, are colored in black and brown, respectively.

To determine whether these deviations are associated with flap opening, RMSD values were recalculated excluding the flap residues (region 43–58). The resulting average RMSD value and shaded error ranges are shown in Figure 3, while individual replica profiles are provided in Supplementary Figure S2.

Average, standard deviation and maximum RMSD values are reported in Table 2, along with the percentage difference between total and “no-flap” RMSD values (see Δ (%) column in Table 2).

As expected, the removal of the flap region leads to a marked reduction in RMSD values in the native system, with average reductions ranging from 25 to 29% across replicas (see Table 2). In the mutants, the reduction is more variable; for example, R3 of L63Q shows only a 2.5% decrease. Notably, even after excluding the flap region, all mutants retain higher RMSD values than the WT, with T12A remaining the most flexible. This indicates that their increased flexibility is not limited to flap motion.

### 2.2. Protein Flexibility

**Figure 4:**
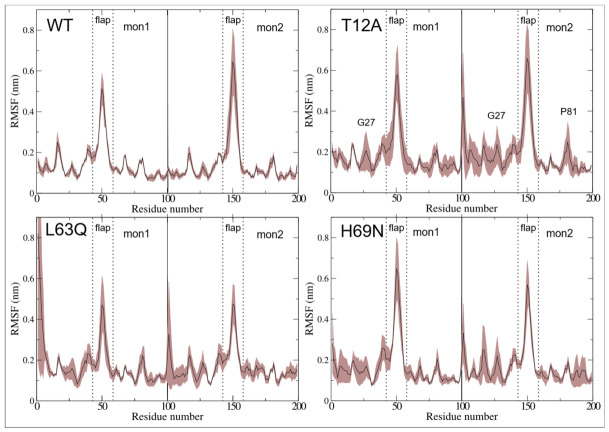
Plot of root-mean-square fluctuations (RMSF) for the two protein chains and the four systems. Average and shaded error range, calculated from the three replicas, are colored in black and brown, respectively.

We next calculated the root-mean-square fluctuation (RMSF) to identify residues whose dynamics are most affected by the mutations. RMSF profiles for the native and mutant systems are shown in Figure 4 as averages with shaded error ranges across replicas, while individual profiles are reported in Supplementary Figure S3.

As expected, the largest fluctuations occur within the flap domains in all systems, across all replicas and both chains. However, notable differences emerge. In the native protein, the two flap domains exhibit asymmetric behavior, with significantly greater fluctuations in chain B than in chain A. In particular, replica R2 (red in Figure S3) shows maximal fluctuations in chain B and minimal fluctuations in chain A compared to R1 and R3 (black and green in Figure S3, respectively). A similar asymmetry is observed in the H69N mutant, although with an inversion between chains: i.e., the R3 replica has maximum fluctuations in chain A and minimum in chain B compared to R1 and R2. In the same system, an important asymmetry of fluctuations is observed also between the two flap domains of R2.

The T12A and L63Q mutations have pronounced but opposite effects. T12A increases flexibility in regions outside the flaps, while flap dynamics remain comparable to WT. This is especially evident in replica R2, where Gly27 (adjacent to the catalytic Asp25) shows enhanced flexibility in both chains (see Figure S3). Additionally, Pro81 in the wall loop exhibits the highest flexibility outside the flap region in chain B. Conversely, the L63Q mutation exerts a stabilizing effect on global motion of PR, with flap fluctuations never exceeding 0.6 nm in any replica or chain.

### 2.3. Open Flap Conformation of the HIV-1 PR Systems

The differences between the RMSD with and without the flap region indicate a strong variability in the motion of this region over the 500 ns and across the three replicas. The importance of this region for PR catalytic activity is also evident from the RMSF plots.

For this reason, we calculated the distance between the c-alpha atoms of Ile50 in monomers 1 and 2 as indicator of the open state of the flaps [20,21,22]. The results show substantial variability in flap opening among replicas and systems. All systems visit open-flap conformations, but only the L63Q mutant does so in less simulation time than the WT, whereas the H69N and T12A distance distributions are shifted toward larger distances, consistent with a higher number of open-flap conformations. Using a 1 nm cut-off, the WT across the three replicas exhibits 65.4% of conformations with distances longer than this threshold, while the corresponding values for L63Q, H69N, and T12A are 55.2%, 82.1%, and 82.6%, respectively. Notably, T12A displays the highest percentage of open-flap conformations, followed by H69N.

To verify that a 500 ns simulation length for the three replicas is sufficient to adequately sample the energy barrier associated with flap opening and closing, we calculated the total number of open–closed and closed–open transitions in each system/replica within the equilibrated 50–450 ns time window. The total numbers are reported in Table 3, considering that all systems started from a closed conformation.

Residence times (τ) were calculated and are reported in Table 3 (in ps) as the average time spent in each state between consecutive transitions, taking into account the percentage of time spent in the open conformation in each trajectory, following standard kinetic analyses of discrete-state trajectories and Markov state model frameworks [23,24]. Despite the high variability among replicas, all residence times are shorter than 1 ns, except for R1 of T12A, which has a τ in the open conformation of 1.645 ns. Although the calculation of highly accurate residence times is beyond the scope of the present study, we conclude that a length of 450 ns per replica is sufficient to reliably sample the opening and closing of the PR flaps.

### 2.4. Secondary Structure

**Figure 5:**
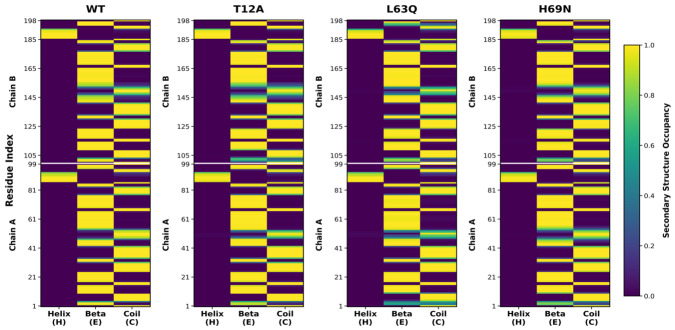
Secondary structure analysis of four systems (WT, T12A, L63Q, and H69N). The plots show residue-wise secondary structure propensities (Helix, β-sheet, and Coil) for chains A and B, averaged over three independent replicas. Colors represent the mean secondary structure occupancy (0–1 scale), highlighting differences in structural stability and local conformational preferences among the variants.

We then compared the secondary-structure content in the four systems. The three simulations show very good reproducibility among the three replicas and conservation of secondary structure throughout the simulations (see Supplementary Figures S4–S15). In Figure 5, we report the secondary-structure occupancy (i.e., the percentage of time spent in a defined secondary structure, averaged over the three simulations). Both native and mutant systems show very good conservation of the alpha-helix around residues 87–95 in both monomers. The nine β-sheets present in each monomer are also conserved over the entire simulation time, with the exception of the four-stranded β-sheet (residues 1–5 and 96–99), which shows reduced structural occupancy only in the three mutants, particularly in L63Q. Notably, the regions harboring the mutations maintain their β-sheet conformation, i.e., the Fulcrum (residues 10–23) in the T12A mutant and the Cantilever (residues 59–75) in the L63Q and H69N mutants. Overall, this analysis indicates that secondary structure remains largely conserved across all variants, and that the observed differences in protein flexibility and flap opening in the mutants are not caused by protein unfolding.

### 2.5. Hydrogen Bonds of the Mutated and Active Site Residues

We then investigated the local perturbations induced by the mutations by analyzing their stable hydrogen bonds (i.e., present for more than 60% of the simulation time). Mutation of Thr12 to alanine reduces the number of stable hydrogen bonds from six (involving residues Ile13, Lys14, Lys20, Glu21, and Cys67) to only one, between the main chain of Ala12 and Arg8. Conversely, mutation of Leu63 to Gln increases the number of stable hydrogen bonds from one (between the main chains of Leu63 and Gly16) to ten, involving residues Gly16, Gly17, Gln61, Ile64, Glu65, Lys70, and Ala71. The H69N variant shows a hydrogen-bond network more similar to that of the native system. In this case, the number of stable hydrogen bonds increases from six to nine. The residues involved in stable hydrogen bonds are Ile66, Ala71, Gln92, and Ile93 with His69 in the WT; Pro1, Ile66, Cys67, Gly68, and Lys70 with Asn69 in the mutant.

We also analyzed the impact of the mutations on the active-site residue Asp25 by examining the stable hydrogen bonds formed by this residue in the four simulated systems. In the WT we observed eleven stable hydrogen bonds between the main and side chain of Asp25 in both monomers, involving residues Arg8, Leu23, Thr26, Gly27, Ala28, and Ile85. In T12A, fourteen hydrogen bonds exceed the 60% simulation-time threshold, between Asp25 and Arg8, Thr26, Gly27, Ala28, and Ile85. In L63Q, ten stable hydrogen bonds are formed between Asp25 and Arg8, Thr26, Gly27, and Ala28. H69N shows thirteen stable hydrogen bonds between Asp25 and Arg8, Leu23, Thr26, Ala28, and Ile85.

All mutations, therefore, preserve key stable interactions between Asp25 and the three residues Arg8, Thr26, and Ala28. Three additional residues form stable interactions with Asp25, but not in all systems: Leu23 in WT and H69N; Gly27 in all systems except H69N; and Ile85 in all systems except L63Q. The nature of these interactions further contributes to the dynamic structure of the active site, with some hydrogen bonds not reaching the 60% threshold because they switch donor or acceptor atoms. For example, the interactions between Arg8 NH2 and Asp25 OD1 and Asp25 OD2 are present for 39% and 27% of the simulation time, respectively.

### 2.6. Long Range Correlated Motions

**Figure 6:**
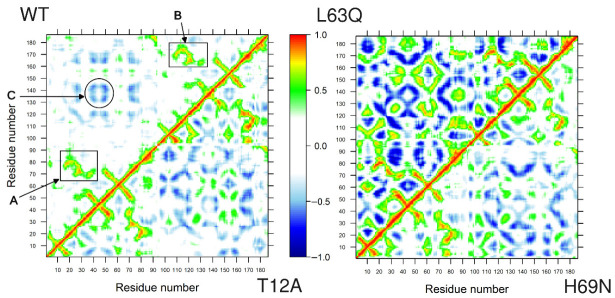
Comparison of dynamic cross-correlation maps for native (upper left triangle) and T12A system (lower right triangle), and L63Q (upper left triangle) and H69N system (lower right triangle). Specific regions are highlighted with rectangles A, B and circle C.

We further investigated long-range interactions in HIV-1 PR by building Dynamic Cross-Correlation Maps (DCCMs) from the concatenated three trajectories (last 450 ns of each replica). Highly positive values of the map elements (Cij) indicate a strong correlation between the motions of residues i and j (colored in green, yellow, and red in Figure 6), whereas negative Cij values denote that the two residues move in opposite directions (anti-correlated motion; colored in cyan, light blue, and dark blue in Figure 6). Both correlated and anti-correlated motions are relevant when investigating biological macromolecules, particularly when residues are located far apart in the protein sequence.

This analysis shows that, in the native protease, long-range correlated motions are established that are functionally linked to the biological role of PR. In particular, strong correlations are observed between residues 12–43 (encompassing the Fulcrum, Fireman’s Grip, and Elbows) and residues 65–90 (corresponding to the Cantilever β-sheet, the Wall, and the α-helix) in monomer 1, and the equivalent regions in monomer 2, highlighted by rectangles A and B, respectively, in Figure 6. These correlated motions connect the active-site region with the central, structured core of PR. In the native system, anti-correlated motions between the two flap regions are also evident, highlighted by circle C in Figure 6.

**Figure 7:**
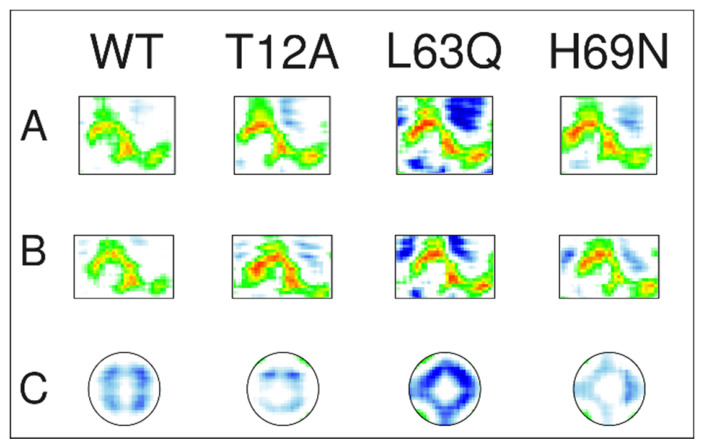
Comparison of the region enclosed in rectangles A, B and circle C in WT of the DCCM of Figure 6 with the corresponding regions in the mutants. Colors as in Figure 6.

To facilitate comparison of the correlated motions highlighted by rectangles A and B in Figure 6 for the WT with the corresponding regions in the mutants, we extracted these regions from the DCCM, together with the correlated motions between the two flaps (circle C in Figure 6), and present them in Figure 7. The pattern of correlated motions in regions A and B is clearly preserved across all systems. Notably, the flap regions, shown in C, consistently exhibit anti-correlated motions, which are more pronounced in L63Q than in the WT, and less pronounced in T12A and H69N.

Overall, the T12A mutation in the Fulcrum and the L63Q and H69N mutations in the Cantilever do not disrupt the native long-range network of correlated motions, but they do introduce a large number of additional correlated and anti-correlated motions in other regions of PR (see Figure 6). This increase is particularly pronounced in the L63Q mutant.

## 3. Discussion

In this molecular dynamics study, we investigated the effects of three mutations (T12A, L63Q, and H69N) on the structure and dynamics of HIV-1 protease. Overall, the results indicate that none of the mutations induces a global structural destabilization of the enzyme, consistent with their natural occurrence.

Notably, the lack of major changes in the overall enzyme structure agrees with previous structural and biochemical studies showing that HIV-1 protease preserves its global fold even in highly drug-resistant variants [6,9]. This highlights the remarkable structural robustness of the enzyme and suggests that resistance is often driven by subtle dynamic changes rather than large-scale structural rearrangements.

All systems preserve their secondary structure throughout the 500 ns simulations and across all replicas (see Figure 5 and Figures S4–S15), as well as key contacts in the active site and the main long-range interdomain correlated motions observed in the wild-type system (see Figure 7). However, despite this apparent structural stability, the mutations markedly modulate the internal dynamics of the protease, particularly affecting flap mobility, hydrogen bond networks, and long-range correlated motions involving the entire protein.

RMSD analyses show that all mutant systems sample conformational states that deviate more from the starting structure than the native protease. This effect is particularly pronounced for T12A, which exhibits consistently higher RMSD values across all replicas (see Figure 2 and Table 1). Importantly, this increase is not solely attributable to flap motion. Even after excluding flap residues from the calculation, mutants still display larger deviations than the native system, indicating that the mutations reshape the global conformational landscape of HIV-1 PR rather than inducing only localized perturbations (see Figure 3 and Table 2).

RMSF analyses further reveal mutation-specific effects on protein flexibility (see Figure 4). As expected, the largest fluctuations occur within the flap domains (residues 43–58) in all systems and both monomers, confirming their intrinsic flexibility and functional relevance. In the native protease, the flap dynamics exhibit pronounced asymmetry between the two monomers, a feature previously associated with functional opening and closing mechanisms [25]. This asymmetry is preserved but reshaped in the mutant systems. A common feature is the anti-correlated motion between the two flaps even though with different magnitude (see C in Figure 7).

In particular, T12A shows increased flexibility in regions outside the flaps, including residues adjacent to the catalytic site (e.g., Gly27), suggesting an enhanced coupling between local active-site dynamics and distal structural regions. In contrast, L63Q exhibits an overall stabilizing effect, with reduced fluctuations both within and outside the flap domains, while H69N displays an intermediate behavior with localized modulation of flexibility.

The functional relevance of these dynamical differences is further supported by the analysis of flap opening, monitored through the inter-monomer distance between the c-alpha atoms of Ile50. All systems sample open-flap conformations, confirming that flap opening is an intrinsic dynamic feature of HIV-1 PR. However, both the frequency and extent of these conformations are mutation-dependent. T12A and H69N exhibit a clear shift toward more open states compared to the native enzyme, whereas L63Q samples open conformations less frequently.

These findings indicate that mutations in the fulcrum (T12A) and cantilever (H69N) regions can enhance flap opening, potentially increasing active-site accessibility, whereas L63Q appears to restrict flap motion. The lack of a simple correlation between flap opening and global RMSD further suggests that the flap dynamics are governed by a complex interplay of local and long-range interactions rather than by isolated structural elements.

Analysis of stable hydrogen bonds shows that each mutation induces a distinct local reorganization of interaction networks without disrupting the catalytic core. T12A markedly reduces the number of stable hydrogen bonds at the mutation site, increasing local flexibility in the fulcrum region. Conversely, L63Q introduces an extensive hydrogen bond network, consistent with its stabilizing effect on both local and global dynamics. H69N produces a hydrogen bonding pattern more similar to the native system, explaining its intermediate behavior.

Despite these local rearrangements, key interactions involving the catalytic residue Asp25 are preserved across all systems. Stable hydrogen bonds between Asp25 and residues such as Arg8, Thr26, and Ala28 are maintained, indicating that the fundamental architecture of the active site remains intact. Thus, the mutations modulate the dynamic environment of the catalytic site without compromising its structural integrity, supporting a mechanism of functional regulation through dynamical tuning rather than structural disruption.

Dynamic cross-correlation analyses provide further insight into the impact of the mutations on global communication pathways within HIV-1 PR. In the native protease, well-defined networks of correlated motions connect the Fulcrum, Hinge, Flap, and Cantilever regions across both monomers, linking the active site to distal structural elements. These long-range correlations are thought to be essential for coordinated flap motion and enzymatic function [20,22,26].

All three mutations preserve the overall framework of correlated motions but introduce additional correlated and anti-correlated movements throughout the protein. This effect is particularly pronounced in L63Q, which displays an expanded network of long-range correlations, consistent with its enhanced structural rigidity and reduced flap mobility.

Taken together, these results indicate that mutations in the fulcrum and cantilever regions of HIV-1 PR act as dynamic modulators rather than structural disruptors. T12A promotes increased conformational variability and enhanced flap opening, potentially facilitating substrate access while altering active-site coupling. L63Q stabilizes the protease by reinforcing hydrogen bond networks and long-range correlations, resulting in reduced flap mobility. H69N exhibits mixed behavior, maintaining native-like hydrogen bonding while shifting the flap dynamics toward more open conformations.

Overall, this mutation-dependent tuning of protein dynamics highlights the critical role of distal regions in regulating HIV-1 PR function and provides a mechanistic framework for understanding how non-active-site mutations can exert long-range effects on protein region involved in enzymatic activity, potentially contributing to drug sensitivity through dynamic modulation rather than direct structural alterations.

## 4. Materials and Methods

### 4.1. Structure Preparation and Mutant Generation

The experimentally determined structure of the Native HIV-1 PR [PDB ID: 1ODW] was retrieved from the RCSB Protein Data Bank (https://www.rcsb.org/) for our study [27]. The ligand di-tert-butyl {iminobis[(2S,3S)-3-hydroxy-1-phenylbutane-4,2-diyl]}biscarbamate (chain C) was removed from the native structure to create a model to use in the MD simulations. Chimera’s Rotamers tool, using Dunbrack backbone-dependent rotamer library, was used to replace the native residues in the HIV-1 protease structure in an equilibrated native MD structure (see following paragraph) and create mutant structures for MD simulations [28]. The residue rotamer with the highest probability (if required) was chosen for each mutant protease model.

### 4.2. Molecular Dynamics Simulations

Topology files for MD simulations were created with the GROMACS gmx pdb2gmx tool [29]. Protonation states of ionizable residues were assigned assuming physiological pH (~7.0). The HIV-1 PR system was added into a cubic box with a minimum distance of 1.0 nm between the protein and the box edges, solvated with water (TIP3P water model) [30] and neutralized with the addition of ions using the GROMACS tools: editconf, solvate and genion, respectively. Charmm 36 force field was used to describe the system during the MD protocol [31]. The protein simulation box was minimized with both the steepest descent algorithm, followed by the conjugate gradient algorithm, until convergence was achieved. After that, 5 ns of MD simulation with GROMACS 2020.3 were produced with a 2 fs time step by applying positional restraints of 1000 kJ mol^−1^ nm^−2^ to the protein atoms. This equilibrated structure was used to create the mutant starting structure, and the same equilibration protocol was used for these systems.

Unrestrained MD simulations of the four systems were carried out with GROMACS 2020.3 for 500 ns each with a 2 fs time step, using the Linear Constraint Solver (Lincs) algorithm [32] on the High-Performance Computing Leonardo system at CINECA center, Bologna, Italy, and using the bioinformatics Elixir-IT resources on g100 at CINECA for the analysis [33].

Long-range electrostatic interactions were treated using the Particle Mesh Ewald (PME) method [34]. During the simulation, a constat temperature of 300 K was maintained using the velocity rescaling thermostat [35], and pressure was kept constant at 1 bar using the Rahman–Parrinello barostat [36].

### 4.3. Analyses of Trajectory Files

To assess trajectory quality and verify convergence of the protein systems toward equilibrium, the root-mean-square deviation (RMSD) was calculated with respect to the first frame of each trajectory, using the GROMACS gmx rms tool. Global stability was evaluated from c-alpha RMSD values over the 500 ns simulations, including calculations performed both with and without the flap region (residues 43–58), in order to distinguish overall conformational changes from flap-specific motions.

Residue-level flexibility was characterized by using RMSF profiles to identify mutation-induced changes in local dynamics, using the GROMACS gmx rmsf tool. Finally, flap opening was monitored by measuring the c-alpha–c-alpha distance between Ile50 residues of the two monomers, and considering the state open for distances greater of 1 nm, allowing quantification of the population of open conformations across replicas and systems. This is the most widely validated metric to define the conformational state of the HIV-1 PR flaps [20,21,22].

Residence times (τ) were calculated as the average time spent in each state between consecutive transitions, following standard kinetic analysis of discrete-state trajectories and Markov-state-model frameworks [23,24], and considering the percentage of time spent in the open state in each trajectory.

The secondary structure analysis was performed using a custom Python v3 script based on the MDAnalysis library [37], which interfaces with the Define Secondary Structure of Proteins (DSSP) algorithm to assign secondary structure elements to each residue along the MD trajectories [38]. For each system (WT, T12A, L63Q, and H69N), DSSP assignments were extracted frame by frame and grouped into three main categories—helix, β-sheet, and coil. The residue-wise secondary structure propensities were then calculated by averaging over the entire simulation time and across three independent replicas, providing a robust comparison of structural stability and local conformational preferences among the variants.

Hydrogen bonds were calculated using GROMACS gmx hbond tools, with standard geometrical criterion: i.e., distance between the donor and the acceptor less than 0.35 nm; hydrogen-donor-acceptor angle minor less than 30°. A hydrogen bond was considered “stable” when present for more than 60% of simulation time. DCCM (Dynamical Cross-Correlation Matrix) were calculated using the dccm() function in the Bio3D R package [39].

## 5. Conclusions

Several classes of antiretroviral drugs are currently available for the treatment of HIV infection, including nucleoside and non-nucleoside reverse transcriptase inhibitors, as well as viral protease inhibitors. The introduction of protease inhibitors into highly active antiretroviral therapy (HAART) has markedly reduced morbidity and mortality, significantly improving the life expectancy of people living with HIV. Understanding the structural and dynamical effects of protease mutations therefore remains essential for elucidating mechanisms that may influence enzymatic function and therapeutic response.

In this molecular dynamics study, we investigated the effects of the T12A, L63Q, and H69N mutations on the structure and dynamics of HIV-1 protease. All variants preserve the global fold, compactness, and secondary structure of the enzyme, indicating that these mutations do not induce significant structural destabilization.

Despite this overall stability, the mutations modulate the internal dynamics of the protease in a mutation-specific manner. T12A and H69N promote increased sampling of open-flap conformations, whereas L63Q reduces flap mobility. These effects are accompanied by distinct rearrangements of local hydrogen bond networks and alterations in long-range correlated motions, while key catalytic interactions involving Asp25 remain conserved.

Overall, the results indicate that mutations in the Fulcrum and Cantilever regions act as dynamic modulators rather than structural disruptors, reshaping the conformational landscape of HIV-1 protease and potentially influencing enzymatic function through changes in protein dynamics.

## Figures and Tables

**Table 1:** Average and standard deviations RMSD for native and mutated PR (nm) in the 50–500 ns time window.

		All C-Alpha		
		Ave	St Dev	Max
Native	R1	0.38	0.051	0.59
	R2	0.36	0.096	0.70
	R3	0.40	0.086	0.75
T12A	R1	0.55	0.051	0.72
	R2	0.62	0.140	1.07
	R3	0.57	0.080	0.78
L63Q	R1	0.45	0.06	0.67
	R2	0.58	0.08	0.84
	R3	0.40	0.09	0.61
H69N	R1	0.51	0.10	0.70
	R2	0.52	0.06	0.71
	R3	0.46	0.08	0.73

**Table 2:** Average and standard deviations RMSD with the exclusion of the flap domains residues (43–58) for native and mutated PR (nm) in the 50–500 ns time window.

		No Flap C-Alpha			
		Ave	St Dev	Max	Δ (%)
Native	R1	0.27	0.03	0.41	−28.95
	R2	0.27	0.04	0.40	−25.00
	R3	0.30	0.05	0.51	−25.00
T12A	R1	0.42	0.03	0.55	−23.64
	R2	0.51	0.11	0.87	−17.74
	R3	0.48	0.07	0.65	−15.79
L63Q	R1	0.39	0.05	0.52	−13.33
	R2	0.46	0.07	0.66	−20.69
	R3	0.39	0.09	0.61	−2.50
H69N	R1	0.43	0.09	0.58	−15.69
	R2	0.41	0.05	0.59	−21.15
	R3	0.33	0.04	0.52	−28.26

**Table 3:** Number of transitions between open and closed conformation of the Flaps, and residence time of the open and closed conformation.

		n. Trans	τ_Open (ps)	τ_Closed (ps)
Native	R1	1077	547	289
	R2	2163	272	144
	R3	2829	208	110
T12A	R1	453	1645	345
	R2	1309	568	120
	R3	3293	226	48
L63Q	R1	3333	75	61
	R2	1005	247	201
	R3	883	281	228
H69N	R1	2387	155	34
	R2	509	726	158
	R3	1255	294	64

## Data Availability

Dataset available on request from the authors.

## Supplementary Materials

The following supporting information can be downloaded at: https://www.mdpi.com/article/10.3390/ijms27093832/s1.

## Abbreviations

AIDS	Acquired Immunodeficiency Syndrome
HAART	Highly active antiretroviral therapy
HIV	Human Immunodeficiency Virus
MD	Molecular dynamics
PDB	Protein Data Bank
PLH	people living with HIV
PR	Protease
RMSD	Root-mean-square deviation
RMSF	Root-mean-square fluctuation
RT	Reverse transcriptase
WT	Wild type
