# Supplementary material for: Unveiling the Impact of Drug-Sensitive Mutations on HIV-1 Protease Dynamics: A Molecular Dynamics Simulation Study of the T12A, L63Q, and H69N Variants
Source: Int J Mol Sci. 2026 Apr 25;27(9):3832. doi: 10.3390/ijms27093832 (PMC13164047; doi:10.3390/ijms27093832)
Supplement: Supplementary file 1 [file ijms-27-03832-s001.zip › ijms-4256337-supplementary.pdf]

Unveiling the Impact of Drug-Sensitive Mutations on HIV-1 Protease Dynamics: A Molecular Dynamics Simulation Study of the T12A, L63Q, and H69N variants

Haythem Srihi, Nabil Abid, Lavinia Fabeni, Caterina Precone, Hélène Déméné, Giovanni Chillemi*

Supplementary Material

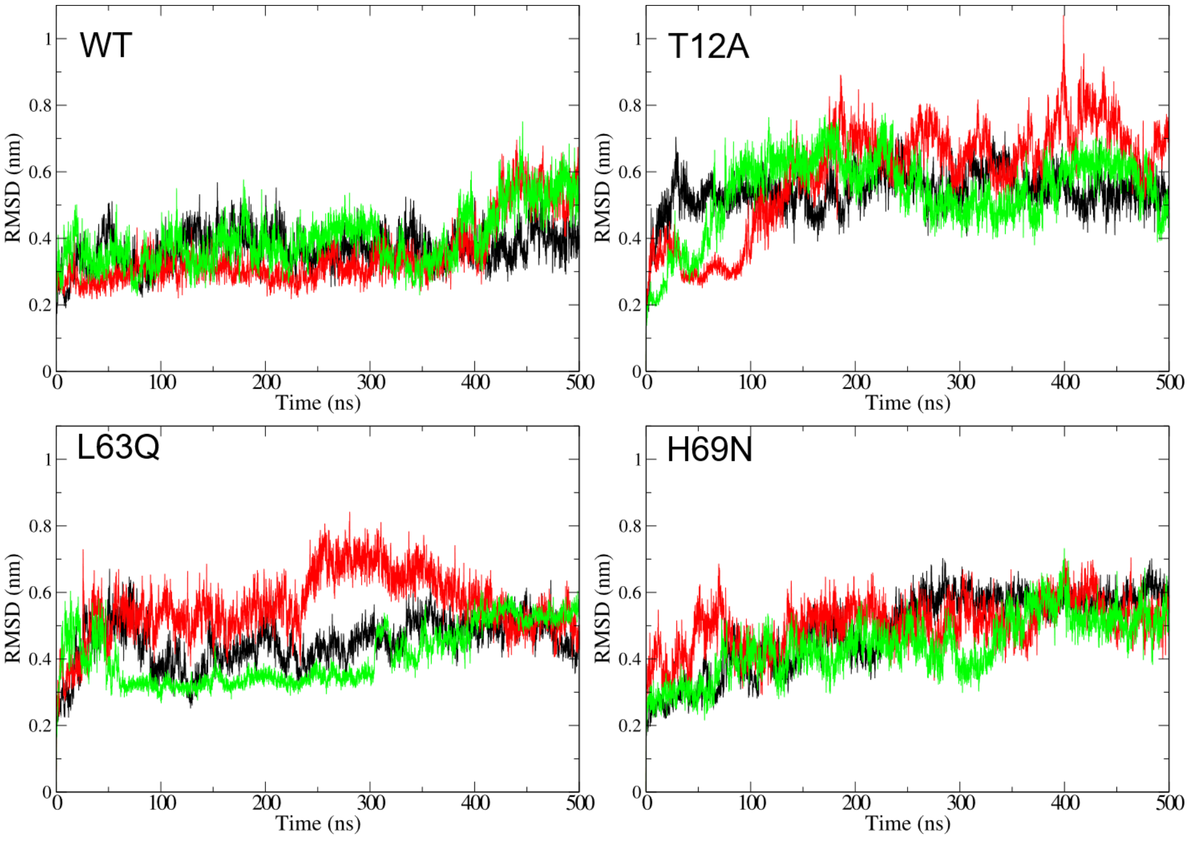

Figure S1. Plot of RMSD of c-alpha atoms, as a function of simulation time for the four simulated PR systems. The three replicas R1, R2 and R3, are colored in black, red and green colors, respectively

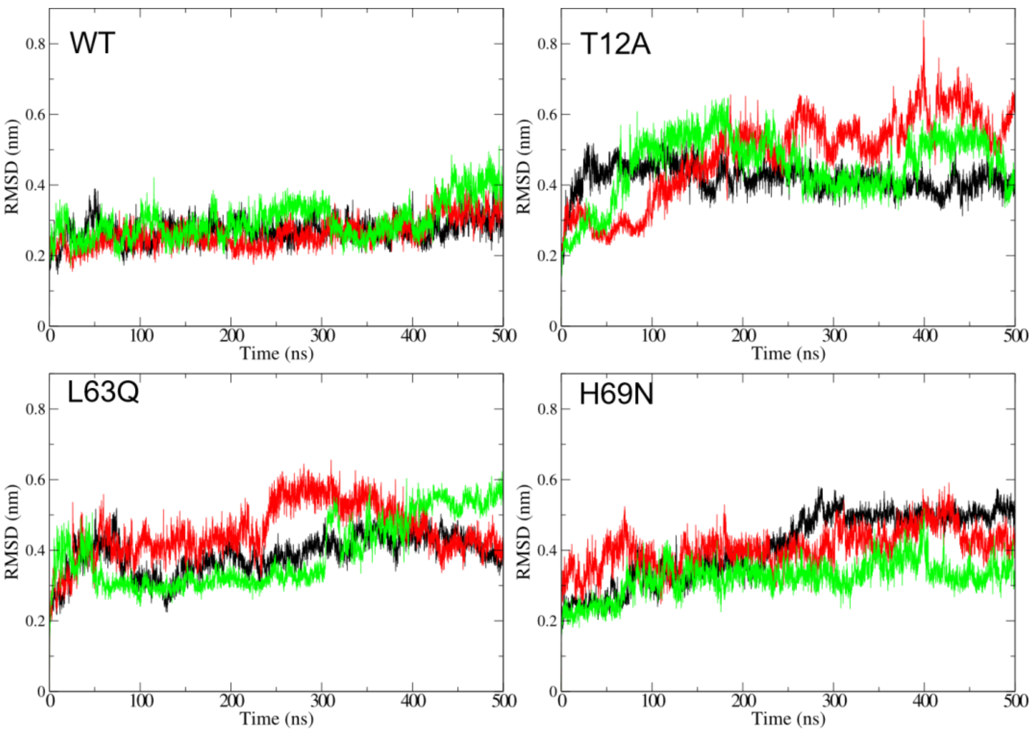

Figure S2. Plot of RMSD of c-alpha atoms with the exclusion of the flap domains residues (43-58), as a function of simulation time for the four simulated PR systems. The three replicas R1, R2 and R3, are colored in black, red and green colors, respectively.

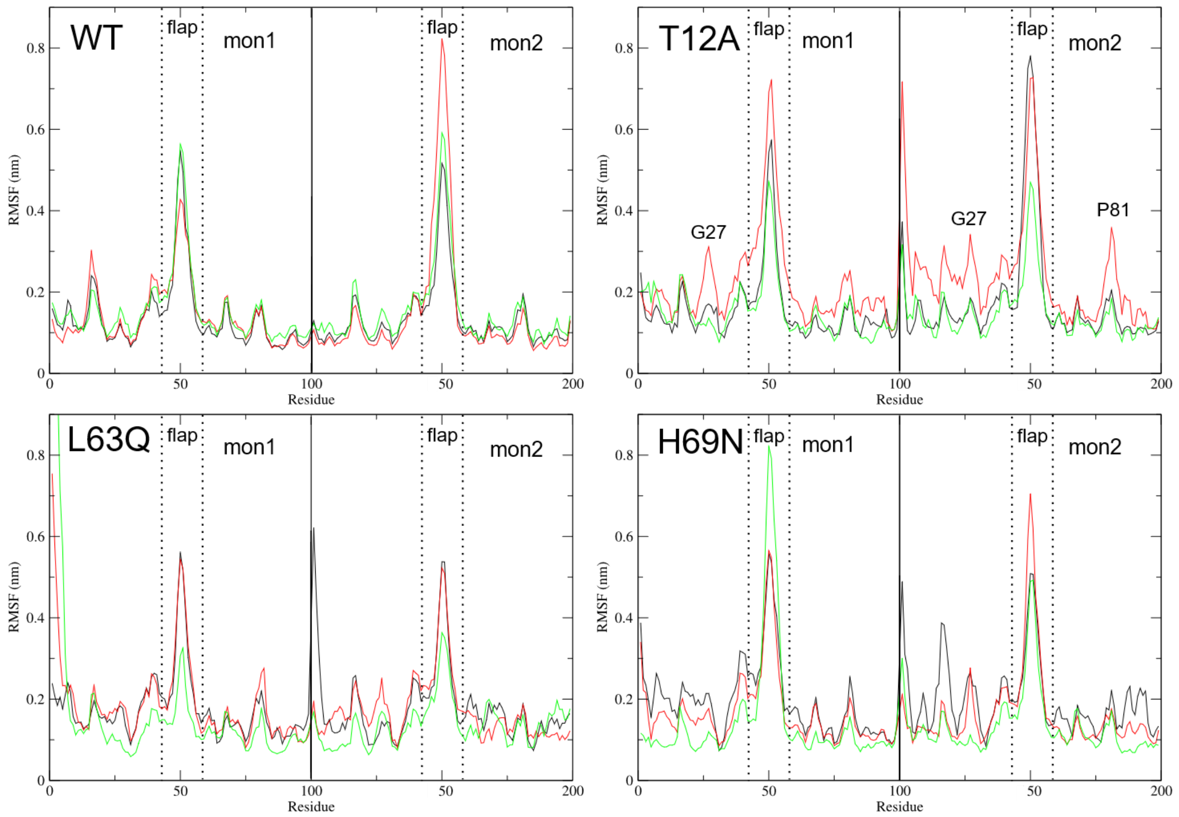

Figure S3. Plot of Root Mean Square Fluctuations (RMSF) for the two protein chains and the four systems. The three replicas R1, R2 and R3, are colored in black, red and green colors, respectively.

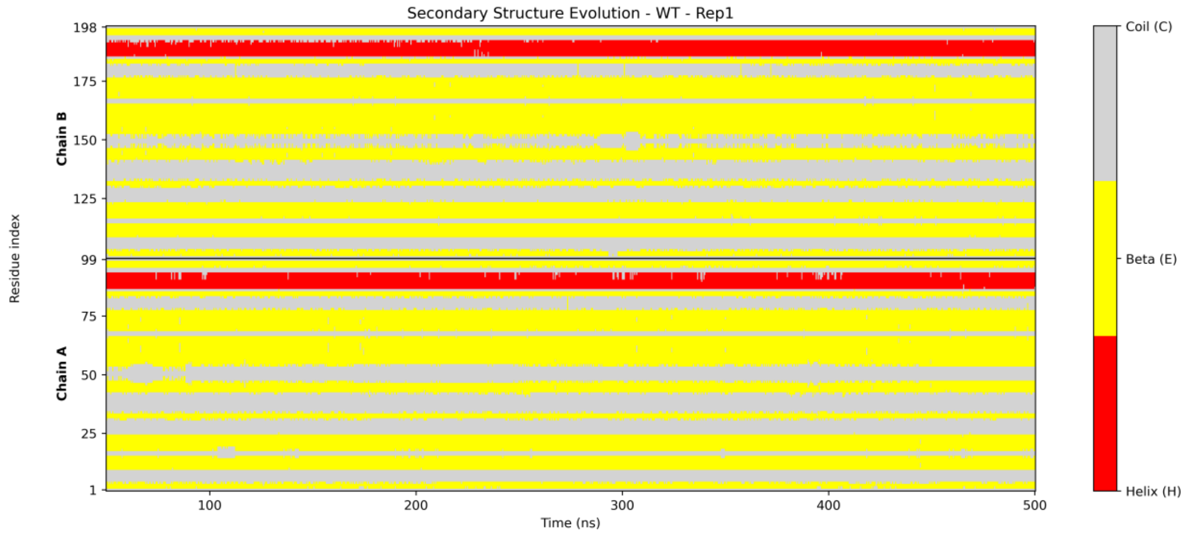

Figure S4. Secondary structure evolution of WT R1 as a function of simulation time. The plot shows the secondary structure assignment for each residue as a function of simulation time, with residues displayed on the y-axis and simulation time (ns) on the x-axis. Chain A (residues 1–99) and Chain B (residues 100–198) are indicated on the left. Secondary structure elements were classified using DSSP and are color-coded as follows: α-helix (red), β-strand (yellow), and coil/loop (gray).

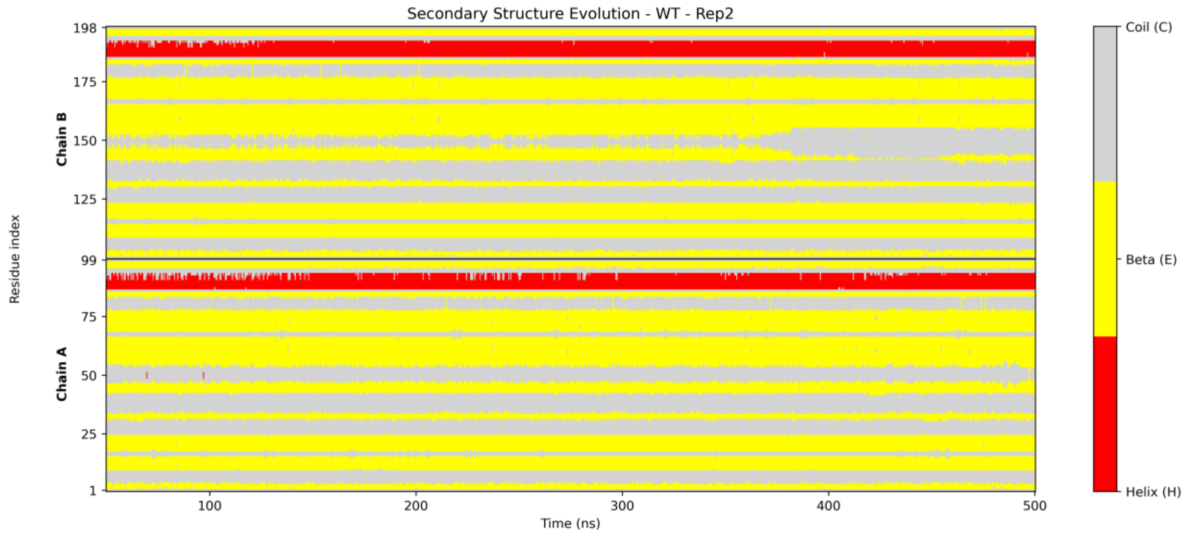

Figure S5. Secondary structure evolution of WT R2 as a function of simulation time. Secondary structure representation and color coding as described in Figure S1.

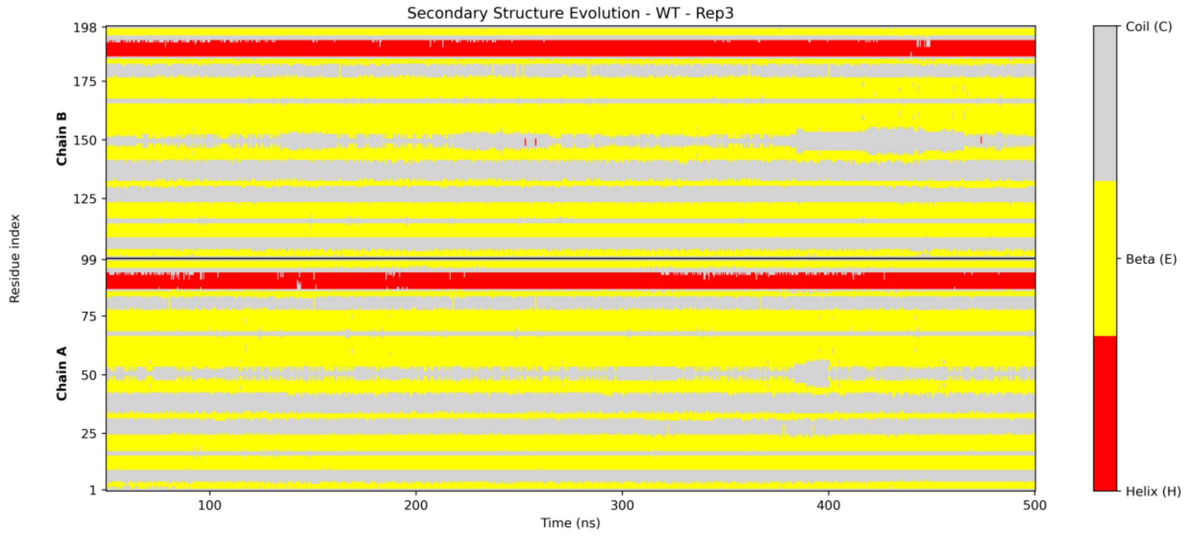

Figure S6. Secondary structure evolution of WT R3 as a function of simulation time. Secondary structure representation and color coding as described in Figure S1.

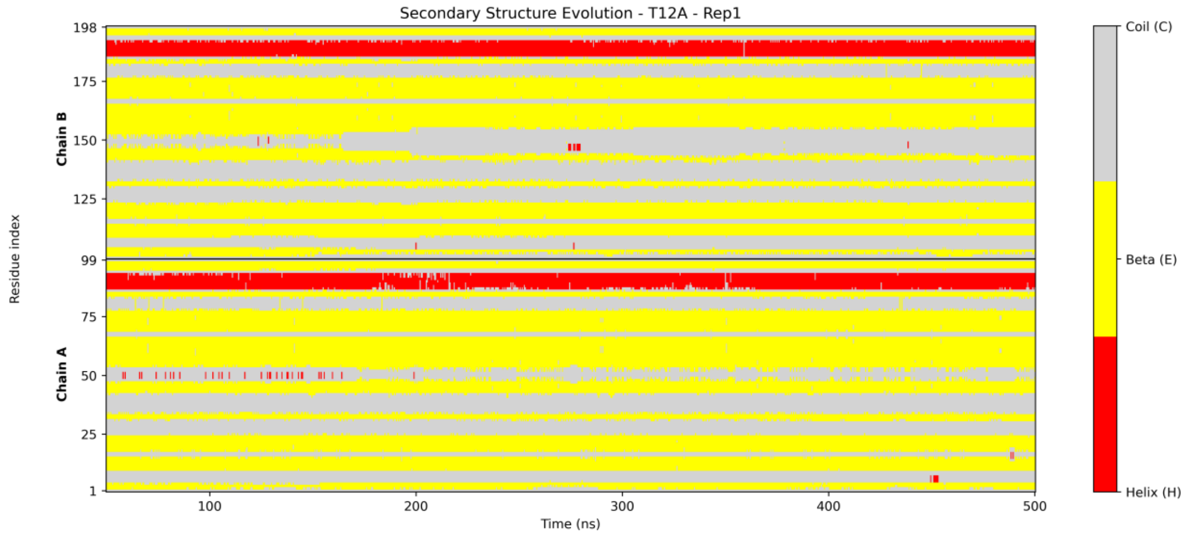

Figure S7. Secondary structure evolution of T12A R1 as a function of simulation time. The plot shows the secondary structure assignment for each residue as a function of simulation time, with residues displayed on the y-axis and simulation time (ns) on the x-axis. Chain A (residues 1–99) and Chain B (residues 100–198) are indicated on the left. Secondary structure elements were classified using DSSP and are color-coded as follows: α-helix (red), β-strand (yellow), and coil/loop (gray).

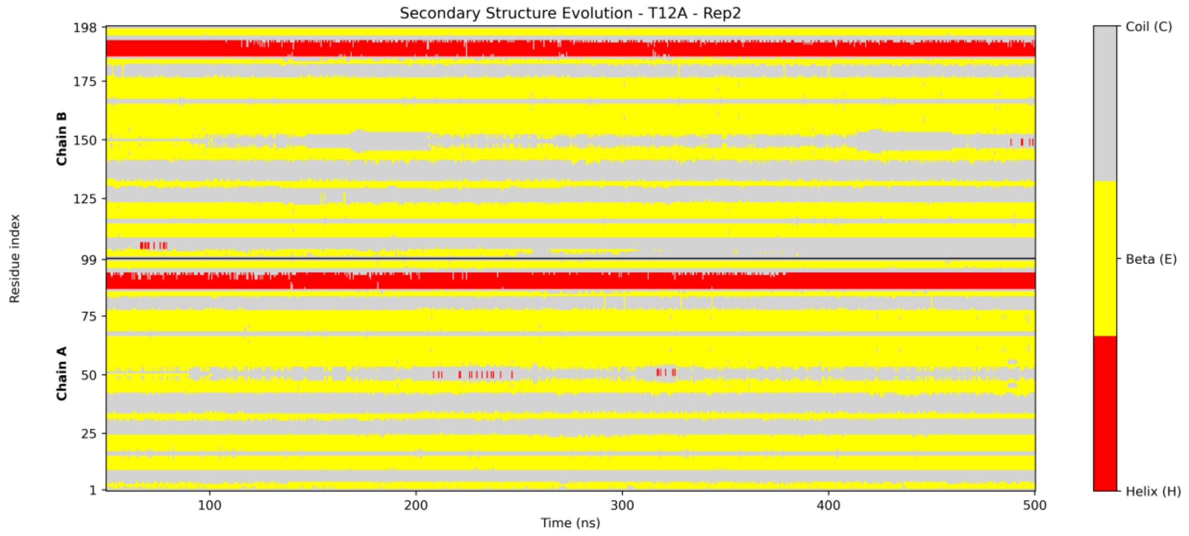

Figure S8. Secondary structure evolution of T12A R2 as a function of simulation time. Secondary structure representation and color coding as described in Figure S4.

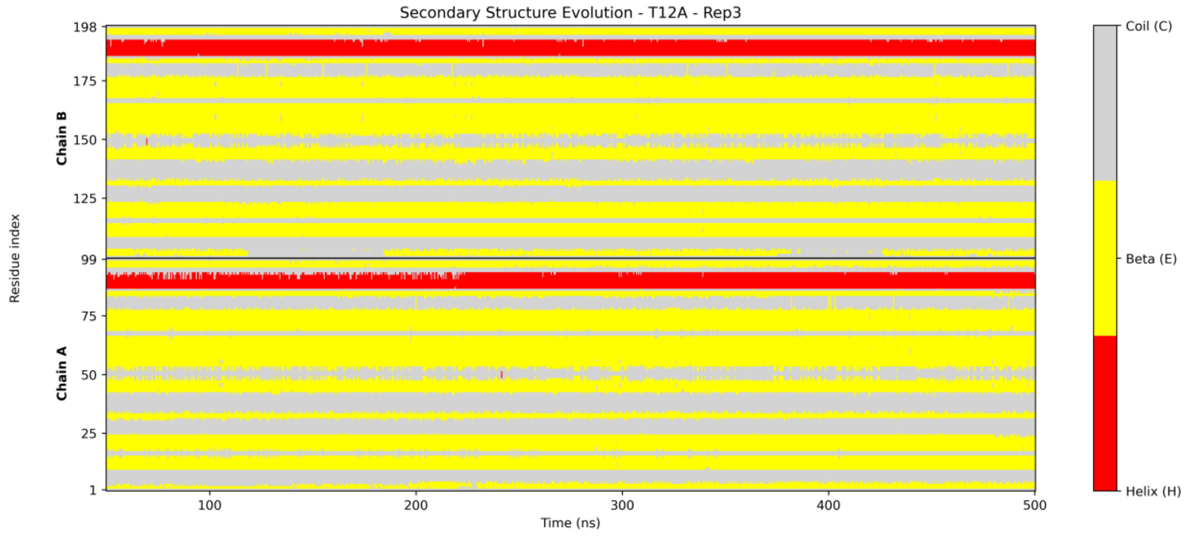

Figure S9. Secondary structure evolution of T12A R3 as a function of simulation time. Secondary structure representation and color coding as described in Figure S4.

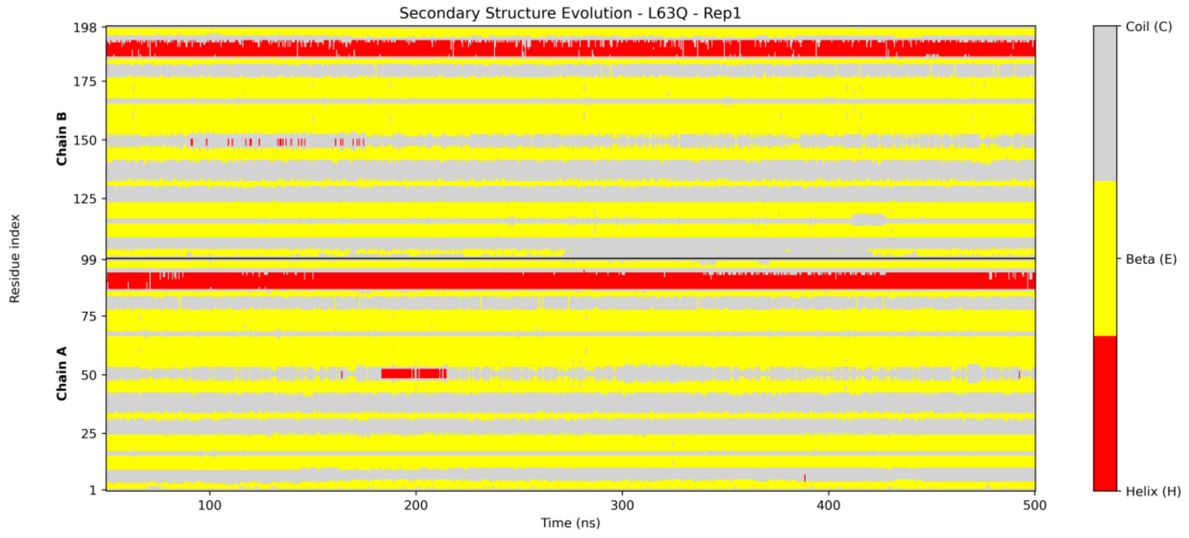

Figure S10. Secondary structure evolution of L63Q R1 as a function of simulation time. The plot shows the secondary structure assignment for each residue as a function of simulation time, with residues displayed on the y-axis and simulation time (ns) on the x-axis. Chain A (residues 1–99) and Chain B (residues 100–198) are indicated on the left. Secondary structure elements were classified using DSSP and are color-coded as follows: α-helix (red), β-strand (yellow), and coil/loop (gray).

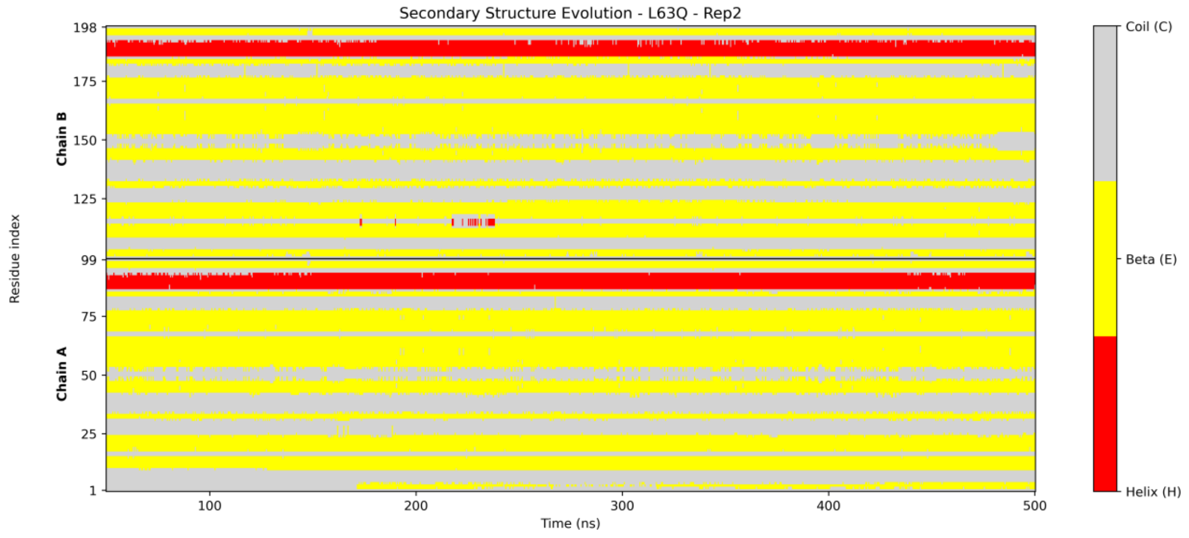

Figure S11. Secondary structure evolution of L63Q R2 as a function of simulation time. Secondary structure representation and color coding as described in Figure S7.

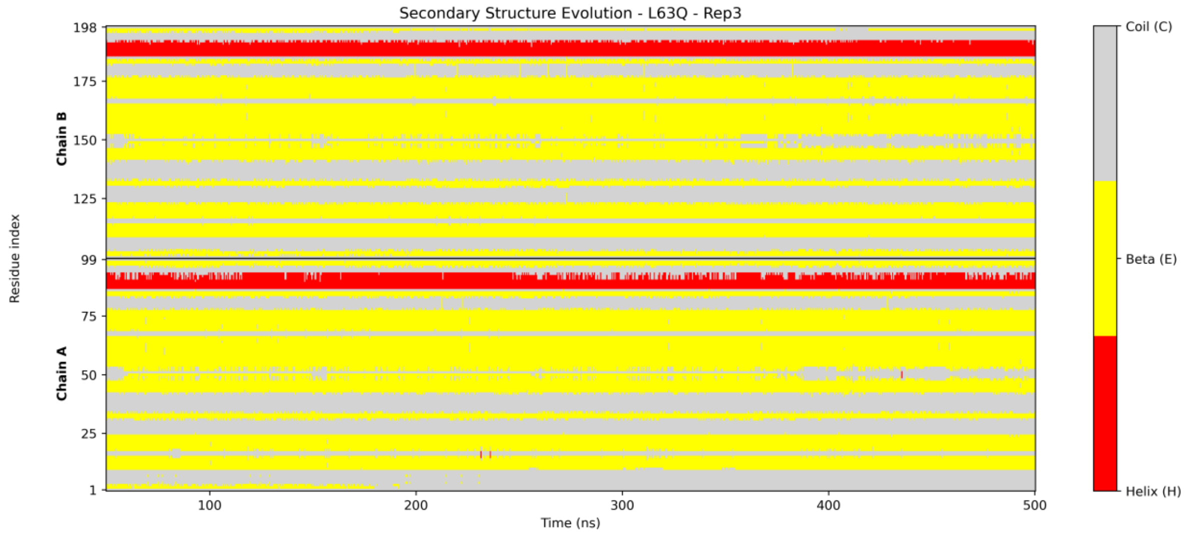

Figure S12. Secondary structure evolution of L63Q R3 as a function of simulation time. Secondary structure representation and color coding as described in Figure S7.

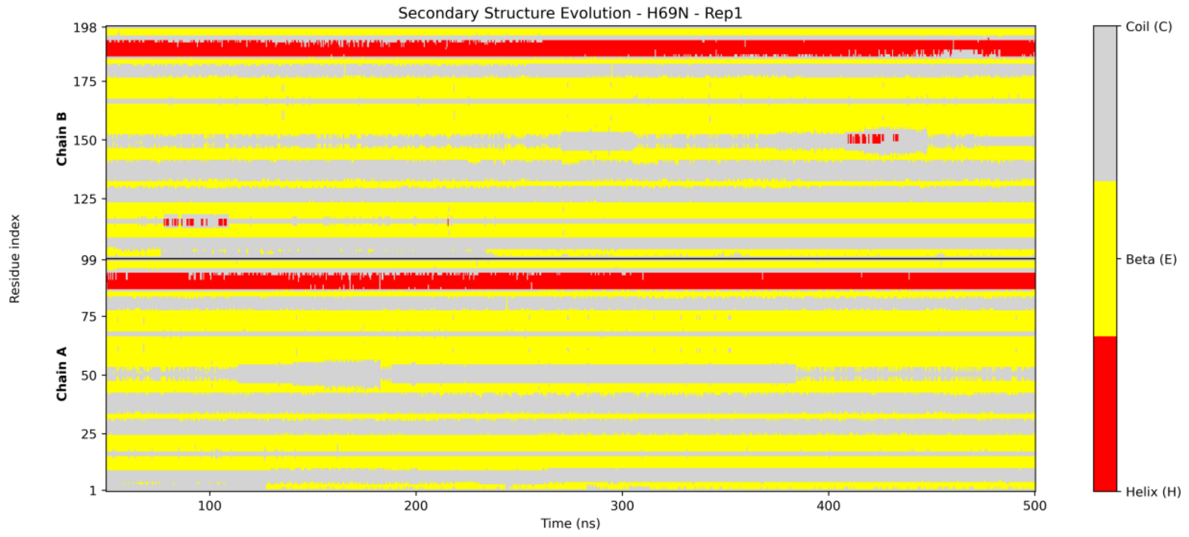

Figure S13. Secondary structure evolution of H69N R1 as a function of simulation time. The plot shows the secondary structure assignment for each residue as a function of simulation time, with residues displayed on the y-axis and simulation time (ns) on the x-axis. Chain A (residues 1–99) and Chain B (residues 100–198) are indicated on the left. Secondary structure elements were classified using DSSP and are color-coded as follows: α-helix (red), β-strand (yellow), and coil/loop (gray).

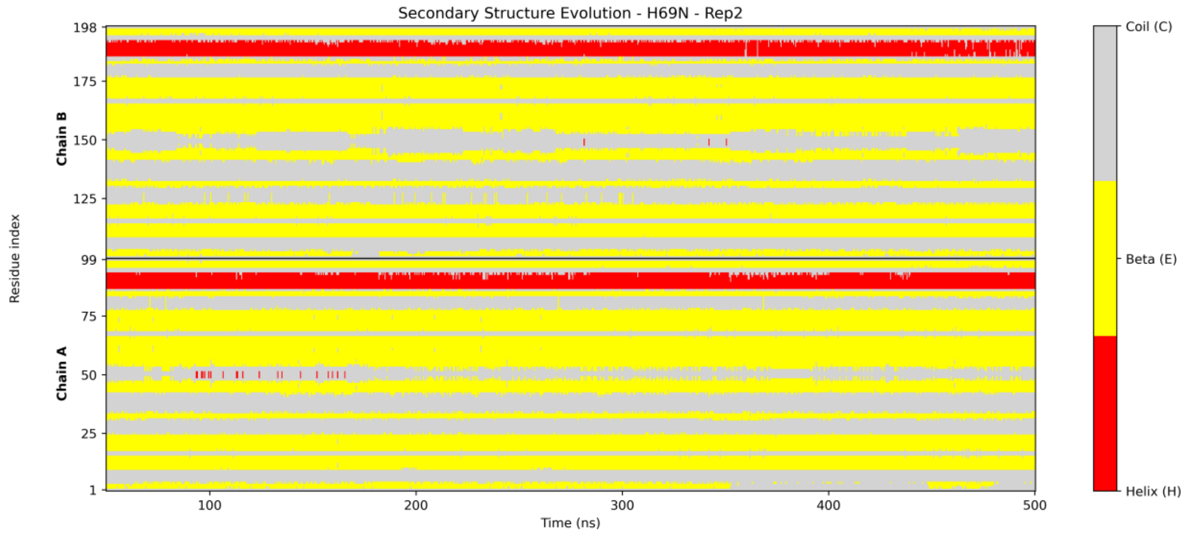

Figure S14. Secondary structure evolution of H69N R2 as a function of simulation time. Secondary structure representation and color coding as described in Figure S10.

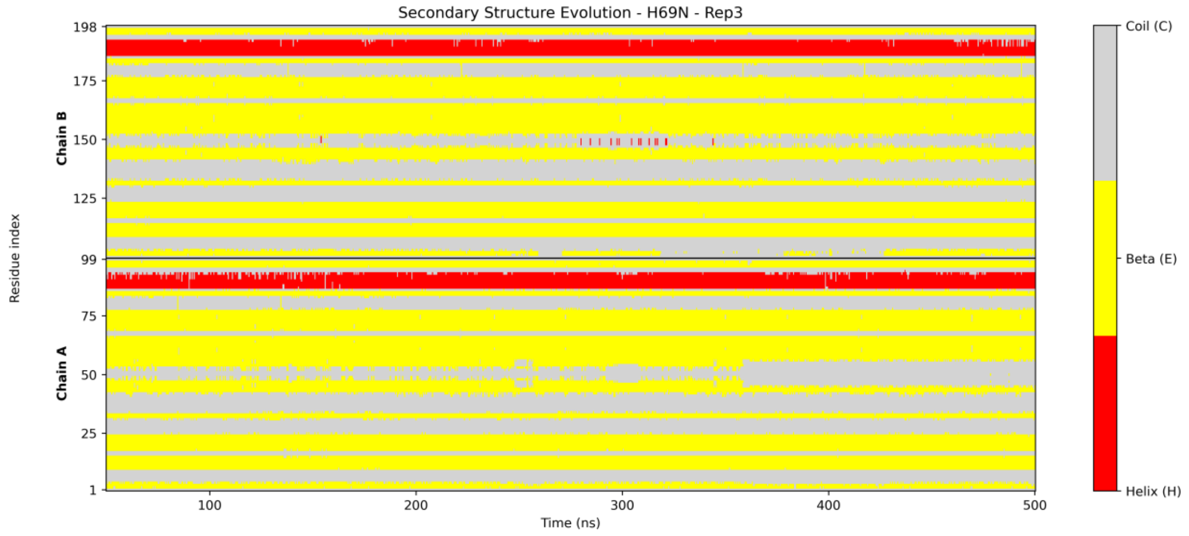

Figure S15. Secondary structure evolution of H69N R3 as a function of simulation time. Secondary structure representation and color coding as described in Figure S10.
